# Differential Transcription of Selected Cytokine and Neuroactive Ligand-receptor Genes in Peripheral Leukocytes from Calves in Response to Cautery Disbudding

**DOI:** 10.3390/ani10071187

**Published:** 2020-07-14

**Authors:** Kavitha Kongara, Venkata Sayoji Rao Dukkipati, Hui Min Tai, Axel Heiser, Alan Murray, James Webster, Craig Brian Johnson

**Affiliations:** 1 School of Veterinary Science, Massey University, Palmerston North 4410, New Zealand; R.Dukkipati@massey.ac.nz (V.S.R.D.); axel.heiser@agresearch.co.nz (A.H.); a.murray@massey.ac.nz (A.M.); C.B.Johnson@massey.ac.nz (C.B.J.); 2 School of Agriculture and Environment, Massey University, Palmerston North 4410, New Zealand; 3 Town and Country Vets, 257 Great South Road, Drury, Auckland 2113, New Zealand; hmin_93@hotmail.com; 4 AgResearch, Hopkirk Research Institute, Palmerston North 4410, New Zealand; 5 AgResearch, Ruakura, Hamilton 3214, New Zealand; jim.webster@agresearch.co.nz

**Keywords:** calves, disbudding, gene transcription, cytokines, pain, meloxicam, nCounter

## Abstract

**Simple Summary:**

Calf disbudding is a painful husbandry practice on dairy and beef cattle farms. Continuing efforts to enhance the accuracy of pain assessment can aid in the application of effective anti-nociceptive (analgesic) agents in non-verbal animals. The aim of this study was to evaluate the changes in the expression of genes involved in inflammation and pain sensitisation in response to removal of horn buds in calves, using hot-iron cauterization. The efficacy of an analgesic, meloxicam, was also tested in attenuating the changes in expression of the studied genes post-disbudding. It was revealed that cautery disbudding induces significant changes in the expression of genes involved in inflammation. Meloxicam was able to blunt the increased expression of some of the genes at 4 h and 24 h after disbudding, while it could not attenuate the increased expression of a few other genes associated with inflammation.

**Abstract:**

Calf disbudding is a painful husbandry practice on dairy and beef cattle farms. An objective measurement of pain is useful to reliably evaluate the pain intensity and anti-nociceptive (analgesic) efficacy of therapeutic agents. The aim of this study was to investigate the changes in peripheral leucocyte inflammatory cytokine gene expression in calves after disbudding, and to assess whether the changes in cytokine gene expression could be an indicator of the efficacy of analgesic drugs. In a randomised controlled study, 16 calves (aged 31 to 41 days and weighing 58 to 73 kg), undergoing routine disbudding, were randomly allocated into two groups (*n* = 8 in each group). Calves in the control group received no analgesic, while those in the treatment group received 0.5 mg kg^−1^ meloxicam subcutaneously prior to disbudding. Disbudding was performed using an electric debudder. Blood (10 mL) was sampled from the jugular vein just before and 4 and 24 h post-disbudding, RNA was extracted from leukocytes, and the transcription of 12 genes of interest was assessed using nCounter gene expression assay. The results showed significantly higher transcription (compared to baseline values) of the studied genes (except *CRH*, *IFNγ*, and *IL10*) in the control group calves at either 4 or 24 h post-disbudding. The administration of meloxicam one hour before disbudding significantly attenuated the upregulation of *IL6*, *PGHS2*, *TAC1*, *NOS1*, and *CRH* gene transcription post-disbudding, while it did not suppress the elevated transcription of acute and pro-inflammatory cytokines such as *IL1β*, *IFNγ*, *IL8*, and *TNFα* genes. In conclusion, nCounter gene expression assay seems to be a promising tool to study the expression of cytokine genes and thus could be used for the pre-clinical evaluation of novel analgesics.

## 1. Introduction

Calf disbudding is a routine management practice on dairy and beef cattle farms. The veterinary medical associations in several countries recommend this procedure to be performed at an early age [1]. Since late 2019, the disbudding of calves in New Zealand requires the administration of a local anaesthetic [2]. However, it is still performed without the provision of analgesia in most countries. Pain is a subjective state, and it can only be measured indirectly in non-verbal animals [3]. Previous studies have used a variety of pain assessment methods following calf disbudding. These include changes in behaviour [4] and/or physiological variables such as plasma cortisol [4,5,6,7], substance P levels [8,9], heart rate, eye temperature [7,8,10], nociceptive thresholds [7,8], and performance measures such as activity and weight gain [11].

During the recent decade, several studies have focussed on evaluating the effect of local anaesthetics and nonsteroidal anti-inflammatory drugs (NSAID) in mitigating the acute pain and distress following the disbudding/dehorning of calves [3,9,11]. The findings of those studies varied regarding the effects of local anaesthetics and/or analgesics on measures of pain and welfare following disbudding in calves. While some studies demonstrated that pre-procedural administration of local anaesthetics and/or analgesics resulted in benefits such as acute pain relief, increased weight gains, and growth rates [3,11], a few other studies did not find any significant reduction of physiological and behavioural changes [8,9,12]. None of the pain assessment methods have been described as the gold standard for measuring the nociceptive blocking ability of analgesics. 

The molecular mechanisms behind nociception and the resultant peripheral and central sensitisation are gradually being unravelled. Cytokines are small proteins secreted by different cell types at the site of injury and by systemic immune cells in response to injury [13]. They appear rapidly following injury due to active gene transcription and translation by the injured cells. They initiate the acute phase response and induce the production of other pro-inflammatory cytokines resulting in amplified response to injury. Increased expression of inflammatory cytokine genes has been demonstrated post-surgery in humans and rodents [14], while perioperative analgesia attenuated the rise in inflammatory cytokine levels and postoperative pain scores in humans [14]. In addition, a significant increase in the expression of inflammatory cytokine mRNA was evident in tissue samples from the testes, epididymis, and scrotum after the castration of calves [15]. Important pro-inflammatory cytokines include interleukin 1 beta (*IL1B*), interleukin 6 (*IL6*), interleukin 8 (*IL8*), tissue necrosis factor α (*TNFα*) and interferon gamma (*IFNγ*) [13], while interleukin 10 (*IL10*) is an anti-inflammatory cytokine, and its induction has been shown to attenuate systemic inflammatory response [16]. Apart from pro-inflammatory cytokines, several molecules involved neuroactive ligand–receptor interaction, such as the nitric oxide synthase 1 (*NOS1*), prostaglandin-endoperoxide synthase 2 (*PGHS2*), angiotensin II receptor type 2 (*AGTR2*), corticotropin-releasing hormone (*CRH*), nerve growth factor (*NGF*), and tachykinin precursor 1 (*TAC1*), have an important role in nociception pathways [17]. Very few studies looked at the expression of inflammatory cytokine genes in response to calf disbudding [18,19]. A precise understanding of the cytokine response to injury can be exploited therapeutically to improve animal welfare.

Quantitative reverse transcriptase polymerase chain reaction (RT-qPCR) is routinely used to investigate gene expression. However, the enzymatic reactions (reverse transcription and polymerization) used in this method could contribute to variability. nCounter gene expression assay enables the amplification-free multiplex detection of nucleic acids by the molecular barcoding of target molecules using a colour-coded probe pair [20]. 

The aim of this study was to investigate the changes in peripheral leucocyte inflammatory cytokine gene expression, using nCounter assay, in calves after disbudding, and to assess whether the changes in cytokine gene expression could be an indicator of the efficacy of analgesic drugs. It was hypothesised that the cautery disbudding of calves would induce significant changes in systemic inflammatory cytokine gene transcription, and that meloxicam (an NSAID analgesic) administered before disbudding would significantly attenuate the changes in cytokine gene expression.

## 2. Materials and Methods

### 2.1. Animals, Groups and Ethics Approval

This trial was undertaken on Holstein–Friesian Jersey crossbred calves born at the AgResearch farm, Tokanui, Hamilton, New Zealand, during the July to September calving season. The study protocol (#15/61) was approved by the Massey University Animal Ethics Committee, Palmerston North, New Zealand. Sixteen calves (aged 31 to 41 days and weighing 58 to 73 kg), undergoing routine disbudding, were randomly allocated into two groups (*n* = 8 in each group). Randomisation was performed by an online software QuickCalcs (GraphPad Software, San Diego, CA, USA). Calves in the control group received no analgesic before disbudding, and those in the treatment group received meloxicam prior to disbudding. During the study period, the calves were housed together indoors in a calf-rearing unit floored with wood shavings and maintained under normal farm practice. 

### 2.2. Disbudding and Blood Sampling

Calves in the treatment group received 0.5 mg kg^−1^ meloxicam (Metacam 20 mg/mL for injection, Boehringer Ingelheim, NZ Ltd., Manukau, New Zealand) subcutaneously (SC) 1 h prior to disbudding. No analgesic was administered to the control group calves. Calves in both groups were sedated with 0.1 mg kg^−1^ Xylazine (Xylazine 2% injection, Phoenix Pharm Distributors, Auckland, New Zealand) intravenously 10 min before disbudding. Disbudding was performed by a single veterinarian in all calves by cautery, using an electric debudder (Shoof International Ltd., Cambridge, New Zealand). The contact time between the cautery iron and each horn bud was maintained for 12–15 s [11]. Blood (10 mL) was sampled from the jugular vein into heparinised vacutainers just before and 4 and 24 h post-disbudding and transported to the laboratory at ambient temperature for processing. The 4 and 24 h sampling time points post-disbudding were chosen with a view to study the kinetics of the early and late transcription of cytokine and neuroactive ligand-receptor genes. Since calves in the control group received no analgesic either prior to disbudding, meloxicam (0.5 mg kg^−1^ SC) was administered soon after the final sampling at 24 h post-disbudding to alleviate pain. 

### 2.3. RNA Extraction and Purification

Two ml of heparinised blood contained in a 50 mL conical tube was mixed with 20 mL pre-warmed tris-buffered ammonium chloride (TAC) buffer (46 mM Tris-Cl, pH 8.1, and 1 mM CaCl_2_), incubated at 37 °C for 10 min, and centrifuged at 350× *g* for 7 min at room temperature. The supernatant was discarded, and the pellet was re-suspended in one mL of RNeasy Lysis Buffer (Qiagen GmbH, Hilden, Germany). RNA in the lysates was purified using a QIAamp RNA Blood Mini Kit (Qiagen GmbH, Hilden, Germany), and its quantity and quality were assessed using a Nanodrop spectrophotometer (Thermo Fisher Scientific Inc., Waltham, MA, USA). RNA concentrations in the samples were adjusted to ≥25 ng/μL and stored at −80 °C. 

### 2.4. Enumeration of Gene-Specific RNA

The detection and enumeration of RNA specific to 15 genes (12 of interest and 3 reference) was performed using nCounter gene expression assay [20]. Forty-eight RNA samples pertaining to the 16 animals, over three time points, were analysed using a custom-designed probe panel for 15 genes. Details of the genes, their Genebank accession numbers, and the position of target sequences are shown in Table 1. The simultaneous detection of mRNA of the 15 genes (Table 1) was undertaken using this assay as per the protocol outlined in the nCounter™ Gene Expression Assay Manual, v.20090807 (NanoString, Seattle, WA, USA). In brief, the gene-specific capture and reporter probes were hybridised to complementary target mRNA (around 100 ng) in solution by overnight incubation at 65 °C in a thermocycler, followed by washing off the excess probes and non-target transcripts in the solution by a two-step magnetic bead-based purification on the robotic nCounter™ Prep Station (NanoString, Seattle, WA, USA). Subsequently, the tripartite molecules were eluted and immobilised on cartridge for the enumeration of different colour-coded probe-mRNA hybrids, using a nCounter™ Digital Analyzer (NanoString, Seattle, WA, USA) with maximal sensitivity (555 fields of view, FOV). 

### 2.5. Processing mRNA Expression Data

Tabulated data in comma-separated value (CSV) format, obtained from the nCounter™ Digital Analyzer as reporter code count (RCC) file, was input into nSolver™ Analysis Software, version 4.0 (https://www.nanostring.com/products/analysis-software/nsolver) and analysed as per the “nCounter Gene Expression Data Analysis Guide”, MAN-C0011-04 (NanoString, Seattle, USA). The reporter library file containing the CodeSet information specific for the genes in this study was used to undertake the quality control routine. Default quality control (QC) settings were used: an Imaging QC (a measure of the percentage of requested fields of view successfully scanned in each cartridge lane) of <75; a Binding Density QC (a measure of reporter probe density in each cartridge lane) range of 0.1 to 2.25; a Positive Control Linearity QC (a measure of correlation between the counts observed for the positive control probes and the concentrations of the spike-in synthetic target nucleic acids) of <0.95; and a Positive Control Limit of Detection QC (indicates whether the counts for the positive control E probe and target sequence spiked in at 0.5 fM, assumed to be the system’s limit of detection, are significantly above the counts of the negative control probes) of ≤2 SD above the mean of the negative controls. All samples (except a 24 h sample for an animal in the meloxicam group) passed the quality control. A background minimisation of counts was undertaken by subtracting the number of counts for the highest negative control +2 SD from all the mRNA counts. Subsequently, a positive control normalisation of RNA counts was then performed using the geometric mean of the 6 positive controls included in the nCounter assay. Finally, a biological normalisation of gene-specific RNA counts was undertaken based on the RNA counts of the chosen three mRNA reference genes (glyceraldehyde 3-phosphate dehydrogenase (*GAPDH*), glucuronidase beta (*GUSB*), and 14-3-3 protein zeta/delta (*YWHAZ*), and the final counts were exported into an Excel workbook. 

### 2.6. Statistical Analyses

Differences in normalised mRNA counts with respect to the 12 genes of interest were tested using a mixed model analysis in SAS^®^ 9.4 (SAS Institute Inc. Cary, NC, USA). The employed model included the fixed effects of group (control versus meloxicam), time (0, 4, and 24 h), and their interaction, and the random effect of animals. An autoregression 1 (AR1) model [21] was used to account for the covariance between the repeated measures within the individuals. In addition, to account for minor differences in the baseline (0 h) mRNA counts within each group, baseline values were considered as a covariate in the mixed model. The normality of residuals of data was checked by Shapiro–Wilk and Anderson–Darling tests using the CAPABILITY Procedure in SAS^®^ 9.4 and the residuals were found to be normally distributed. Probability (*p*) values of ≤0.05 were considered statistically significant.

*A priori* power analysis could not be undertaken since there were no prior studies in cattle that used nCounter gene expression assay for pain-related cytokines. Hence, an indicative post-hoc power analysis was performed to estimate the power of detecting a significant difference between the two groups at a given time point as well as between two time points within a group. Power was estimated for two genes (IL8 and PGHS2) that exhibited significant differential expression between groups at 4 and 24 h post-disbudding, respectively. Power was calculated using G*Power software [22], assuming a simple t-test for differences between two independent means. The mean and standard deviation of mRNA counts, as well as sample size of the groups, were used as input values to estimate the realised power. The level of significance was set at 5%. Based on the observed effect size, the number of individuals required to achieve power values up to 1 were extrapolated for each of those two genes. Similarly, using the same software, the power of detecting a significant difference in mRNA counts (compared to baseline values) of *IL8* and *PGHS2* genes at 4 and 24 h, respectively, was also determined. A paired t-test for differences between two dependent means (matched pairs) was assumed. The observed mean and standard deviation of mRNA counts at the two time points as well as sample size were inputted, and a correlation of 0.5 between the readings at the two time points was assumed. 

## 3. Results

**Figure 1:**
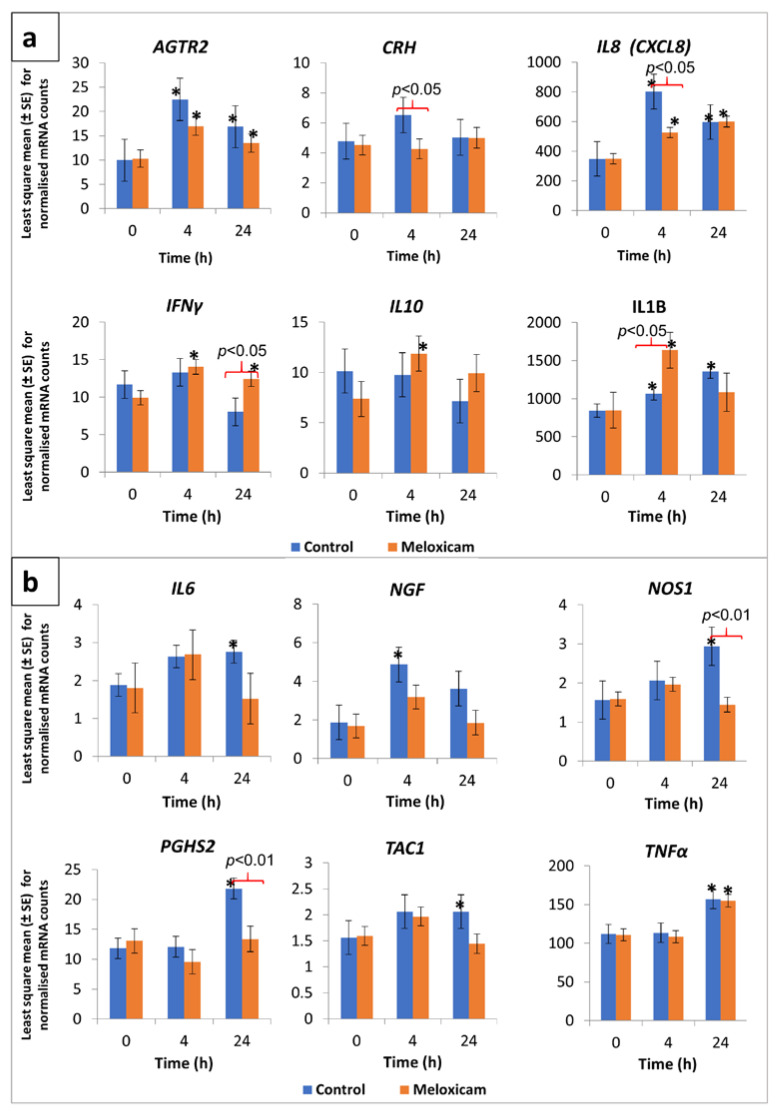
Transcription profile of selected cytokine and neuroactive ligand-receptor genes in peripheral leukocytes from calves, in response to cautery disbudding. Two groups of calves (aged 31 to 41 days, n = 8 per group), that were either administered meloxicam or no analgesic (control), were disbudded using an electric debudder. mRNA counts in peripheral leukocytes, as determined by amplification-free nCounter gene expression assay (NanoString, Seattle, WA, USA), were normalised based on the RNA counts of three reference genes included in the assay. An asterisk (*) over the mean bars indicates a significant (p < 0.05) difference of transcription compared with respective pre-disbudding mean, within treatment, while significant differences between the treatment and control group means, within each time point, were denoted with braces and respective p-values on top of the bars. (**a**) Data pertaining to angiotensin II receptor type 2 (ATGR2), corticotropin-releasing hormone (CRH), interleukin 8 (IL8), interferon gamma (IFNγ), interleukin 10 (IL10), and interleukin 1 beta (IL1B) are shown in this graph. (**b**) Data pertaining to interleukin 6 (IL6), nerve growth factor (NGF), nitric oxide synthase 1 (NOS1), prostaglandin-endoperoxide synthase 2 (PGHS2), tachykinin precursor 1 (TAC1), and tissue necrosis factor α (TNFα) are shown in this graph.

Least square means ± standard errors (LSM ± SE) for normalised mRNA counts in peripheral leukocytes with respect to the 12 genes of interest in control and meloxicam-administered calves, prior to as well as at 4 and 24 h post-disbudding, are presented in Figure 1a,b. Significant differences in mRNA counts, between groups as well as between time points, were observed with regard to *IL8*, *IFNγ*, *IL1B*, *NOS1*, and *PGHS2* genes. The *IL8* gene exhibited significantly higher transcription (compared to baseline mRNA counts) at 4 and 24 h post-disbudding in both groups (Figure 1a). The *IL8* mRNA counts were particularly high in the control group calves at 4 h post-disbudding, which significantly differed from those in the meloxicam-administered calves. *IL1B* transcription was also significantly higher post-disbudding in both groups, with the mRNA counts at 4 and 24 h in the control group and at 4 h in the meloxicam group being significantly higher compared to the respective baseline values (Figure 1a). Furthermore, the *IL1B* mRNA counts at 4 h post-disbudding in the meloxicam group were significantly higher compared to those in the control group. 

A slight but significant increase in *IFNγ* gene transcription, compared to time point 0, was evident in the meloxicam group at 4 and 24 h post-disbudding (Figure 1a). In addition, the mRNA counts for this gene at 24 h post-disbudding in the meloxicam group were significantly higher than those in the control group. The mRNA counts for *NOS1* and *PGHS2* genes were significantly higher in the control group at 24 h post-disbudding compared to their respective baseline values as well as the meloxicam-administered group (Figure 1b).

A significantly higher transcription, compared to respective group baseline mRNA counts, was observed in the case of *AGTR2* (in meloxicam as well as control groups at both 4 and 24 h post-disbudding), *IL10* (at 4 h in meloxicam group), *IL6* (at 24 h in control group), *NGF* (at 4 h in control group), *TAC1* (at 24 h in control group), and *TNFα* (at 24 h in both control and meloxicam groups) genes (Figure 1a,b). In the case of the *CRH* gene, although the mRNA counts were relatively low, a between-group difference was observed at 4 h post-disbudding (Figure 1a). The mRNA counts for this gene in the control group calves were significantly higher compared to the meloxicam-administered calves. 

Post-hoc power analysis results indicated a power of 0.706 for detecting a significant difference in the *IL8* mRNA counts between the meloxicam and control groups at 4 h post-disbudding, and 0.922 in the case of *PGHS2* mRNA counts between the two groups at 24 h post-disbudding. A sample size of 10 and 16 animals per group would increase the power of detection to 0.8 and 0.9, respectively, in case of the *IL8* gene. Similarly, powers of 0.966 and 0.999 were realised in the case of detecting a within-group (control) difference (compared to baseline values) in *IL8* mRNA at 4 h and *PGHS2* mRNA at 24 h post-disbudding, respectively. 

## 4. Discussion

Peripheral leukocyte inflammatory cytokine gene expression profiles were investigated in calves in response to cautery disbudding in this study. Both control and meloxicam groups showed significant changes in a variety of pro- and anti-inflammatory cytokine mRNA after disbudding. There was a significantly higher transcription of *TNFα* gene at 24 h post-disbudding in both groups, but no significant difference was detected at 4 h after disbudding, compared to respective baseline values. *TNFα* is mainly secreted by activated macrophages after tissue injury and plays a pivotal role in the initiation of acute phase response along with other early pro-inflammatory cytokines such as *IL1B*, *IL6*, and *IFNγ* [23]. Disbudding with hot iron involves the burning of a ring of tissue containing horn bud cells. The systemic inflammatory response following a burn injury in mice has been demonstrated to induce the release of a myriad of pro- and anti-inflammatory cytokines, with a peak of serum *TNFα* detected at 24 h and 48 h post-burn [24]. Only a few studies [18,19] have assessed the expression of cytokines and other inflammatory mediators in response to calf disbudding. In the present study, it is likely that the mRNA levels of *TNFα* have started rising in the window between 4 and 24 h and by 24 h after disbudding, a significant increase from the basal level was found. Nonsteroidal anti-inflammatory drugs have been shown to upregulate the production of *TNFα* from human peripheral blood leucocytes [25,26]. Results from the current study appear to support this as the *TNFα* mRNA of meloxicam group, similar to the control group, significantly increased from baseline by 24 h post injury.

*IL1B*, *TNFα*, and *IFNγ* have been postulated to be specific and early markers of inflammation and nociception. No significant differences in *IL1B*, *TNFα*, and *IL6* mRNA abundance between sham-handled and disbudded calves treated with a lidocaine block and IV meloxicam have been reported in one study [18]. In the present study, the mRNA transcripts of both *IL1B* and *INFγ* were found to be significantly elevated in the meloxicam group post-disbudding, compared to the respective baseline values and values of the control group at 4 h and 24 h. A previous study in calves reported a similar elevation of these early pro-inflammatory cytokine levels as early as 15 min after disbudding [19]. In the current study, it is likely that the mRNA of these pro-inflammatory cytokines had begun to rise at an earlier time point than 4 h, which was the first sampling time point after disbudding in our study. The finding that meloxicam did not attenuate the rise in early pro-inflammatory cytokine mRNA in this study appears to support the previous literature that the administration of only a systemic NSAID without local anaesthetic prior to disbudding/dehorning did not completely mitigate the acute phase response [9,19,27].

Calves in both groups showed a significantly elevated transcription of the *IL8* gene at 4 and 24 h post-disbudding (Figure 1a). *IL8* is a pro-inflammatory cytokine produced by a variety of tissues and blood cells in response to inflammation, and it exhibits chemotaxic activities against neutrophils and T lymphocytes, drawing these cells to the site of inflammation [28]. *IL8* has been shown to evoke hyperalgesia in rats by a prostaglandin-independent mechanism [29]. More recently, plasma *IL8* levels were found to be positively correlated with the intensity of burning mouth syndrome pain in humans [30]. In the current study, the significantly lowered transcription levels of this gene in the meloxicam group compared to those in the control group at 4 h post-disbudding potentially indicate the ability of meloxicam to reduce the pain caused by noxious stimuli via its anti-inflammatory effect. This is supported by the observed significantly higher transcription of the *IL10* gene in the meloxicam group at this time point (Figure 1a). In the current study, the significantly lowered transcription levels of this gene in the meloxicam group compared to those in the control group at 4 h post-disbudding potentially indicate the ability of meloxicam to reduce the pain caused by noxious stimuli via its anti-inflammatory effect. This is supported by the observed significantly higher transcription of *IL10* gene in the meloxicam group at this time point (Figure 1a). A similar negative regulatory effect of *IL10* on *IL8* expression in human monocytes has been documented [31]. It is interesting to see markedly elevated transcription levels of *IL8* in the leukocytes of calves in both groups prior to disbudding. This could be due to the age of the calves, as it has been found that *IL8* levels in healthy infant humans are significantly higher than those in adults [32]. The high *IL8* levels in infants have been attributed to be a major T cell effector function that has the potential to activate antimicrobial neutrophils and γδ T cells. 

*IL6* is a Janus-faced complex cytokine. It is one of the pro-inflammatory cytokines released early in the cascade [33] and induces the production of acute phase proteins. It also acts as an anti-inflammatory cytokine. *IL6* production has been shown to be significantly upregulated in skin cells close to a heat-induced injury site in rats at 24 h post-injury [34]. The anti-inflammatory effect of IL6 is thought to be mediated, in part, through the induction of prostaglandin E2 (PGE_2_) synthesis, which in turn leads to the inhibition of *TNFα* and *IL1* receptors and subsequently their production [35,36]. In the present study, *IL6* mRNA counts did not significantly change in the meloxicam group, but the control group exhibited a significant increase at 24 h after disbudding. The precise time point at which the anti-inflammatory effect of *IL6* initiates in the course of inflammation relative to tissue injury is unknown. Treatment with NSAIDs for 3–6 days after injury in humans has been shown to result in lesser concentrations of serum *IL6* compared to a control group [37]. Thus, it appears that meloxicam had stabilized the *IL6* mRNA levels compared to the control group post-disbudding in the current study.

In the present study, the mRNA counts of *PGHS2*, *NOS1*, substance P (*TAC1*), *CRH*, and *NGF* showed no significant differences in transcription between pre-and post-disbudding time points in the meloxicam group. The control group showed a significant increase in the mRNA counts of these neuroactive ligand–receptor interaction molecules at 24 h post-disbudding. The *PGHS2* (aka cyclooxygenase 2, cox-2) gene encodes the inducible isozyme cox-2, which is predominantly involved in inflammatory prostanoid biosynthesis in response to injury [38]. Meloxicam is a preferential cox-2 inhibitor [39] and thus inhibits inflammatory prostanoids to produce its therapeutic effects in injured animals. Our finding of lower *PGHS2* mRNA counts in the meloxicam group could possibly reflect decreased ex vivo prostaglandin E2 synthesis in the plasma after cautery dehorning [40]. 

*TAC1* (a precursor of Substance P, SP) is a neuropeptide member of the tachykinin family and is widely distributed in the central, peripheral, and enteric nervous systems. Neuronal SP is released from sensory neurons on noxious stimulation and activates several immune cells such as macrophages, mast cells, and T lymphocytes [41]. Sensory neuropeptide activated immune cells release inflammatory mediators such as histamine, arachidonic acid derivatives, and cytokines/chemokines. The synergistic interplay between SP and prostaglandins has been demonstrated in various inflammation and pain models [42]. Lower plasma SP concentrations compared with a control group have been reported in meloxicam-treated calves following scoop dehorning without local anaesthesia [9], which is in line with the lower mRNA counts of *TAC1* in the meloxicam group at 24 h after disbudding in our study. 

Nitric oxide synthases (*NOS1*) catalyse the oxidation of the amino acid L-arginine to produce the free radical, nitric oxide (NO). Nitric oxide has been shown to activate the cox enzymes for prostanoid biosynthesis [43] and facilitate prostaglandin-induced hyperalgesia in rats [44]. The inducible isoform of NOS (iNOS) is released from macrophages in response to inflammatory stimuli [45]. Nonsteroidal anti-inflammatory drugs have been shown to inhibit the expression of iNOS mRNA in rat in vitro studies [45]. Likewise, the co-administration of meloxicam and iNOS inhibitor produced a synergistic anti-inflammatory effect in carrageenan-induced acute inflammation in rats [46]. The findings from the current study support those of previous studies demonstrating the efficacy of NSAIDs in inhibiting the NO synthesis, with significantly lower mRNA of *NOS1* in the meloxicam-treated calves compared to the control calves. 

The *CRH* gene encodes a member of the corticotropin-releasing hormone family and is mainly expressed by the hypothalamic paraventricular nucleus and secreted into the hypophyseal portal system [47]. It plays a crucial role in eliciting the stress response through stimulation of the hypothalamic, pituitary, adrenal axis and the secretion of cortisol in response to noxious stimulation [48]. In the present study, post-disbudding *CRH* mRNA were significantly higher in the control group than the meloxicam group at 4 h, and no difference was found in both groups at 24 h after disbudding. Plasma cortisol concentration peaks within 30 min and returns to pre-treatment levels 6–8 h after cautery disbudding [6]. It is likely that more obvious changes in *CRH* mRNA have occurred earlier than 4 h post-disbudding, and only a slight increase could be detected in the control group at 4 h in the current study.

*NGF*, a member of the neurotrophin family, is essential for the development and maintenance of both central and peripheral nervous systems [49]. Rats injected with *NGF* into paws showed rapid and prolonged hypersensitivity to noxious thermal stimulation, confirming its role in inflammatory pain [50]. This inflammatory hyperalgesic effect of *NGF* is thought to be primarily mediated via tropomyosin receptor kinase A (TrkA) receptors [51]. In the current study, it is interesting to note that while there was a significantly higher transcription of NGF in the control group calves at 4 h post-disbudding, its transcription in meloxicam-administered calves at 4 and 24 h after disbudding remained relatively unchanged compared to baseline values. 

Two recent studies [52,53] revealed the *AGTR2* to be a promising target for therapeutics aimed at treating neuropathic pain. The studies showed that damage to the peripheral nerve might lead to pain hypersensitivity as a result of signalling through *AGTR2* found on peripheral macrophages infiltrating the site of injury, rather than those on sensory neurons. The activation of *AGTR2* on peripheral macrophages triggers the release of reactive oxygen/nitrogen, which in turn activates the transient receptor potential ankyrin 1 ion channel, thus leading to nociceptive signaling in sensory neurons [52]. The current study also revealed the significantly higher transcription of this gene at 4 and 24 h post-disbudding in the calves of both groups (Figure 1a), indicating that meloxicam administration could not attenuate the transcription of this gene in the peripheral macrophages. 

*IL10* is a potent anti-inflammatory cytokine, reducing the expression of pro-inflammatory cytokines to balance the inflammatory response to injury [54]. A significant increase in *IL10* gene expression has been reported 12 h after the burdizzo castration of cattle [15]. The pre-emptive administration of an NSAID, carprofen, has been shown to have no effect on *IL10* mRNA expression [55]. Meloxicam has been shown to have no effect on the production of *IL10* from bovine lymphocytes [56] despite the involvement of cox-2 (target of meloxicam) in the production of *IL10* [57]. Similarly, in the current study, meloxicam did not affect the upregulation of IL10 mRNA. Taken together, it appears that NSAIDs such as meloxicam and carprofen do not inhibit cox-2 activity to the extent of suppression of different populations of immune cells that produce *IL10* [56].

In the current study, a representative *post-hoc* power analysis pertaining to the mRNA data of two genes, *IL8* (at 4 h) and *PGHS2* (at 24 h), revealed that there was adequate power (>0.9, except for between-group differences in the case of *IL8* at 4 h, which was 0.71) of detecting between-group and between time-point differences. In the case of *IL8*, a sample size of 10 per group would provide 0.8 power for between-group comparison. It is to be noted that these power analyses were based on assuming a simple t-test, but for the actual analysis of the study data, a more power linear mixed model, which would better account for correlation between repeated measures, was employed. 

It has been shown in humans [58] that cytokine increases in cerebrospinal fluid (CSF) during peripheral surgery are more marked compared to those in blood, indicating the role of pro-inflammatory cytokines in increased central nervous system sensitivity to surgical pain. Hence, to check if a similar trend was evident in the current study, a 1–2 mL CSF was obtained from the atlanto-occipital joint of all calves prior to and 4 and 24 h post-disbudding. However, attempts to quantify the transcription of cytokine and neuroactive ligand-receptor genes (as well as housekeeping genes) in the RNA purified from those samples were unsuccessful due to the very low yield of RNA (<20 ng/sample) being inadequate for the employed nCounter gene expression assay. A volume of 8–10 mL of CSF might contain an adequate number of cells, yielding the required mRNA quantity for the nCounter gene expression assay. 

nCounter gene expression assay (NanoString, Seattle, WA, USA) has been employed in this study to explore the amplification-free expression of peripheral leukocyte inflammatory cytokine genes. The transcription of genes, as actual mRNA counts in relation to the inbuilt positive controls as well as the three selected reference genes (*GAPDH, GUSB, and YWHAZ*) was successfully quantified. Although the expression of only 12 genes (plus 3 reference genes) was investigated in the current study, the expression of as many as 96 genes (of choice) in total could be simultaneously investigated using this assay. Using this technique, differential transcription of a few cytokine and neuroactive ligand-receptor genes was detected in the peripheral leucocytes of claves post-disbudding. Disbudding resulted in an increased transcription of pro-inflammatory cytokine genes (such as *IL1β*, *IFNγ*, *IL8*, and *TNFα*) in all calves (control as well as meloxicam groups), while meloxicam administration attenuated the upregulation of a few other genes (*IL6*, *PGHS2*, *TAC1*, *NOS1*, and *CRH*) involved in pain sensitisation pathways. These findings indicate that meloxicam alone would not be able to completely reduce the pro-inflammatory response following cautery disbudding. However, the nCounter assay seems to be an efficient tool to screen combinations of different analgesics for their ability to attenuate the pro-inflammatory response of a variety of genes. However, further studies need to be undertaken to validate the findings of this study. It would be interesting to see if this differential transcription of genes reflects in terms of actual protein levels, using MILLIPLEX^®^ Cytokine/Chemokine panels (EMD Millipore Corporation, Billerica, MA, USA) that can simultaneously quantify up to 15 different cytokines. Similarly, corroboration of the differential expression of cytokines with either behaviour-based pain scores or other objective methods such as electroencephalogram variables would be useful.

## 5. Conclusions

Hot iron disbudding induced significant changes in the expression of a complex network of inflammatory cytokine mRNA in the peripheral blood leukocytes of calves. The subcutaneous administration of meloxicam one hour before disbudding significantly attenuated the upregulation of IL6, PGHS2, TAC1, NOS1, and CRH gene transcription post-disbudding. The mRNA expression levels of acute and specific pro-inflammatory cytokines such as IL1β, IFNγ, IL8, and TNFα were significantly increased after disbudding, and meloxicam did not suppress the elevation of these cytokine mRNA compared to the control group. The current study results indicate that the administration of only a systemic NSAID could not completely reduce the acute inflammatory response following disbudding. nCounter gene expression assay (NanoString, Seattle, WA, USA) was used in the current study, which is a promising tool to study the expression of cytokine genes and thus could be used for the pre-clinical evaluation of novel analgesics. However, it would be useful to corroborate these gene transcription results at protein levels in further studies.

## Figures and Tables

**Table 1:** Details of genes analysed for transcription in peripheral leukocytes.

Gene	Gene Category	Genebank Accession	Target Sequence Position
*IFNγ*	Pro-inflammatory cytokine	NM_174086.1	503–602
*IL1B*	Pro-inflammatory cytokine	NM_174093.1	331–430
*IL6*	Pro-inflammatory cytokine	NM_173923.2	293–392
*IL8 (CXCL8)*	Pro-inflammatory cytokine	NM_173925.2	278–377
*TNFα*	Pro-inflammatory cytokine	NM_173966.2	950–1049
*IL10*	Anti-inflammatory cytokine	NM_174088.1	145–244
*AGTR2*	Neuroactive ligand-receptor	XM_001249373.2	1206–1305
*CRH*	Neuroactive ligand-receptor	NM_001013400.1	443–542
*NGF*	Neuroactive ligand-receptor	NM_001099362.1	558–657
*NOS1*	Neuroactive ligand-receptor	XM_867630.5	2657–2756
*PGHS2*	Neuroactive ligand-receptor	NM_174445.2	881–980
*TAC1*	Neuroactive ligand-receptor	NM_174193.1	317–416
*GAPDH*	Reference	NM_001034034.1	213–312
*GUSB*	Reference	NM_001083436.1	1815–1914
*YWHAZ*	Reference	NM_174814.2	147–246
